# The Roles of Inflammasomes in Host Defense against *Mycobacterium tuberculosis*

**DOI:** 10.3390/pathogens10020120

**Published:** 2021-01-25

**Authors:** Jialu Ma, Shasha Zhao, Xiao Gao, Rui Wang, Juan Liu, Xiangmei Zhou, Yang Zhou

**Affiliations:** 1 College of Veterinary Medicine, Southwest University, Chongqing 402460, China; mjl0698@163.com (J.M.); 15087307880@139.com (S.Z.); gaoxiaoyouxiangya@163.com (X.G.); wr07100914@163.com (R.W.); liujuanb@163.com (J.L.); 2 Immunology Research Center, Medical Research Institute, Southwest University, Chongqing 402460, China; 3 State Key Laboratories for Agrobiotechnology, Key Laboratory of Animal Epidemiology of the Ministry of Agriculture, National Animal Transmissible Spongiform Encephalopathy Laboratory, College of Veterinary Medicine, China Agricultural University, Beijing 100193, China; zhouxm@cau.edu.cn

**Keywords:** *Mycobacterium tuberculosis*, inflammasome, NLRP3, AIM2, IFN

## Abstract

*Mycobacterium tuberculosis* (MTB) infection is characterized by granulomatous lung lesions and systemic inflammatory responses during active disease. Inflammasome activation is involved in regulation of inflammation. Inflammasomes are multiprotein complexes serving a platform for activation of caspase-1, which cleaves the proinflammatory cytokines such as interleukin-1β (IL-1β) and IL-18 into their active forms. These cytokines play an essential role in MTB control. MTB infection triggers activation of the nucleotide-binding domain, leucine-rich-repeat containing family, pyrin domain-containing 3 (NLRP3) and absent in melanoma 2 (AIM2) inflammasomes in vitro, but only AIM2 and apoptosis-associated speck-like protein containing a caspase-activation recruitment domain (ASC), rather than NLRP3 or caspase-1, favor host survival and restriction of mycobacterial replication in vivo. Interferons (IFNs) inhibits MTB-induced inflammasome activation and IL-1 signaling. In this review, we focus on activation and regulation of the NLRP3 and AIM2 inflammasomes after exposure to MTB, as well as the effect of inflammasome activation on host defense against the infection.

## 1. Introduction

Despite the development of chemotherapy and vaccine programs, tuberculosis (TB) continues to lead to increasing death tolls and poses a serious threat to global public health [1]. It is one of the top 10 causes of mortality and the leading cause from a single infectious pathogen. WHO estimated that 1.5 million people died from TB in 2018 (https://www.who.int/news-room/fact-sheets/detail/tuberculosis). Approximately one-third of the world’s population is infected with MTB, the main causative agent of TB, and 5-10% of the population develops active TB [2]. MTB can infect the host for decades without causing clinical manifestations, only to reactivate in compromised immunity. Bacterial replication results in a robust granulomatous inflammatory response in immunocompromised patients. Inflammation is indispensable for initial control of infection, and also helps disseminate MTB to susceptible individuals in the community [3]

IL-1β and IL-18, members of IL-1 family, are potent proinflammatory cytokines [4,5,6]. They play a critical role in host defense against MTB infection. Mice deficient in IL-1β or IL-1 receptor type I (IL-1R1) have been shown to be highly susceptible to infection with MTB, as reflected by decreased survival time, increased bacterial burden in lungs and bronchoalveolar lavage fluid (BALF) and extensive pulmonary necrosis [7,8]. IL-18 deficiency in mice elicits higher bacterial burden in lung tissues and larger granulomas in the lungs and spleens. Administering exogenous recombinant IL-18 subcutaneously to IL-18-disrupted mice reduces the sizes of the granulomatous lesions and bacterial load [9]. IL-1β activity is rigorously controlled both at the transcriptional and post-translational levels. IL-1β and IL-18 are synthesized as biologically inactive intracellular precursors which are mainly dependent on nuclear factor-κB (NF-κB) pathway. Then the precursors are cleaved into the bioactive forms by active caspase-1 [10,11] or other enzymes such as proteinase-3 (PR3) [12,13], neutrophil elastase [14,15], cathepsin G [16] and matrix metalloproteinases (MMPs) [17,18]. Caspase-1 activation is caused by assembly of inflammasome, which is a multiprotein platform for processing and secretion of proinflammatory cytokines as well as initiation of pyroptosis [19]. Inflammasomes play a critical role in host defense against pathogens. However, aberrant activation is detrimental, causing tissue damage and even higher mortality. In this review, we discuss the interaction of MTB with inflammasomes and the roles in host defense against the bacteria.

## 2. Events in MTB-Infected Phagocytes

After it is inhaled as aerosolized droplet nuclei and deposited in the distal alveoli, MTB encounters phagocytes in the infected lungs, including alveolar macrophages, dendritic cells (DCs) and neutrophils [1]. A variety of receptors in phagocytes including C-type lectin receptors (CLRs) [20,21,22], scavenger receptors [23] and complement receptors [24], are involved in the binding and uptake of MTB, which are critical for bacterial spread and dissemination. MTB prevents phagolysosome formation via selective exclusion of Rab7, a small GTP binding protein associated with late endosomal compartment, retention of Rab5, which regulates early endosome fusion [25,26], and phosphatidylinositol-3-phosphate (PI3P) production on the phagosomal membranes [27]. Apart from phagosome maturation arrest, the early secretory antigenic target 6 (ESAT-6) system 1 (ESX-1) type VII secretion system and phthiocerol dimycocerosates of MTB act in concert to mediate phagosomal [28,29] or phagolysosomal rupture [30], facilitating its translocation to the cytosol. Restriction of phagosome acidification mediated by vacuolar proton ATPase is necessary for mycobacterial phagosomal rupture [31,32]. Phagosomal rupture leads to host cell death, promoting bacterial spread to new cells [33]. Manipulation of phagosome affects NLRP3 inflammasome activation [34]. 

Granulomas are the classical pathological changes in TB. They represent a stalemate between bacterium and host, preventing bacterial spreading to surrounding healthy tissues and also avoiding bacterial eradication. ASC, the adaptor protein of inflammasomes, is required for granuloma formation. ASC deletion results in fewer granulomas, causing a decreased survival of mice following infection with MTB [35]. A model utilizing transparent zebrafish embryos and GFP-labeled *Mycobacterium marinum* (a close genetic relative of MTB) provided insight into the initiation of granulomas formation. After embryos were injected with *M. marinum*, the bacteria were engulfed by blood macrophages immediately, and extravasated into diverse tissues by 1 day postinfection, leading to bacterial transfer between macrophages. The infected macrophages began to form granuloma-like aggregates at day 3 postinfection. The aggregates mainly consisted of macrophages, epithelioid cells (differentiated macrophages), and multinucleated giant cells (also called giant Langhans cells) [36]. Macrophages are enough to induce granuloma formation at the early stage [37]. Granuloma formation is induced by mycobacterial proinflammatory phosphatidyl-myo-inositol mannosides, lipomannans and anti-inflammatory lipoarabinomannan. Lipomannans contributes to the fusion of granuloma macrophages into multinucleated giant cells, which is mediated by Toll-like receptor 2 (TLR2) and dependent on the β1 integrin/ADAM9 (a disintegrin and metalloprotease domain 9) cell fusion machinery [38]. In addition to macrophages, granulomas also contain dendritic cells [39] and lymphocytes [40]. MTB infection triggers activation of proinflammatory signals [41] as well as production of chemokine [42] and adhesion molecules [43], which facilitate leukocyte recruitment to inflammatory sites [44].

## 3. A Brief Introduction to Inflammasome

Inflammasomes, major components of the innate immune system, consist of sensor proteins, ASC that is not necessary for all inflammasomes such as the NLRP1 and NLRC4 inflammasomes, and executor caspase-1. The sensors interacts with ASC and caspase-1 following detecting pathogen-associated molecular patterns (PAMPs) and danger-associated molecular patterns (DAMPs), leading to assembly of inflammasomes and activation of caspase-1 [45]. Active caspase-1 mediates maturation and release of proinflammatory cytokines such as IL-1β and IL-18 as well as pyroptosis, a programmed necrotic cell death which is mediated via gasdermin D’s membrane pore-forming activity [46,47]. Among inflammasomes, the NLRP3 and AIM2 inflammasomes are extensively described. Upon exposure to chemically- and structurally-unrelated agonists, NLRP3 is activated via its association with mitochondrion-derived molecules, such as cardiolipin [48] and mitochondrial DNA (mtDNA) [49]. Its activation requires two signals: signal I contributes to upregulation of IL-1β and NLRP3 in an NF-κB-dependent manner, which is called priming, and signal II induces activation of the NLRP3 inflammasome characterized by maturation of IL-1β and caspase-1 [50]. Reactive oxygen species (ROS) production controls the priming step in most circumstances [51]. Several organelles, including mitochondria [52], endoplasmic reticulum [53], mitochondria-associated ER membranes (MAMs) [53] and the Golgi apparatus [54,55], participate in NLRP3 inflammasome activation. AIM2 senses non-sequence-specific DNA via electrostatic attraction between the double-stranded DNA (dsDNA) sugar-phosphate backbone and the positively charged hematopoietic expression, interferon-inducible nature and nuclear localization (HIN) domain residues [56,57] oligomerizes at multiple binding sites in dsDNA [58] and recruits ASC and caspase-1 to assemble the AIM2 inflammasome [59].

## 4. MTB and the NLRP3 Inflammasome

Many reports have documented that infection with MTB triggers NLRP3 inflammasome activation in vitro. MTB infection activates the NLRP3 inflammasome in several cell types, including THP-1 monocyte-derived macrophages [60], primary human macrophages derived from peripheral blood mononuclear cells [61], murine bone marrow-derived macrophages (BMDMs) [62], bone marrow-derived dendritic cells (BMDCs) [63], murine retinal pigment epitheliums [64] and primary murine microglial cells [65], from 6 hours postinfection (hpi) [63] to 24 hpi [34,62], based on the fact that maturation of caspase-1 and release of IL-1β are suppressed in cells isolated from Nlrp3^-/-^, Asc^-/-^ or caspase-1^-/-^ mice [63], or after inhibition with lentivirus-mediated shRNA [35,60], siRNA [61] or inhibitors (Ac-YVAD-fmk [60] or VX765 [34] for caspase-1, MCC950 for NLRP3 [34]). MTB H37Rv is more efficient in invading type II alveolar epithelial cells than H37Ra [66]. MTB Infection leads to release of proinflammatory cytokines, including IL-8, IL-6 and TNF-α in A549 alveolar epithelial cells [67]. It is debatable that MTB activates the NLRP3 inflammasome in RAW264.7 [68] because ASC, a necessary component for the NLRP3 inflammasome, is absent in this cell line [69,70]. NLRP3 inflammasome activation restricts mycobacterial growth in macrophages. Caspase-1 overexpression represses mycobacterial growth in THP-1 macrophages [60]. Human monocyte–derived macrophages from patients harboring genetic variants in NLRP3 and CARD8 secret higher levels of IL-1β [71] and display increased MTB growth control [72]. In addition to MTB, other mycobacterium pathogens, including *Mycobacterium kansasii* [73], *Mycobacterium abscessus* [74] and *M. marinum* [75], are also able to trigger NLRP3 inflammasome activation, while attenuated vaccine strain *Mycobacterium bovis* bacillus Calmette–Guérin (BCG) fails to activate NLRP3 [63].

**Figure 1:**
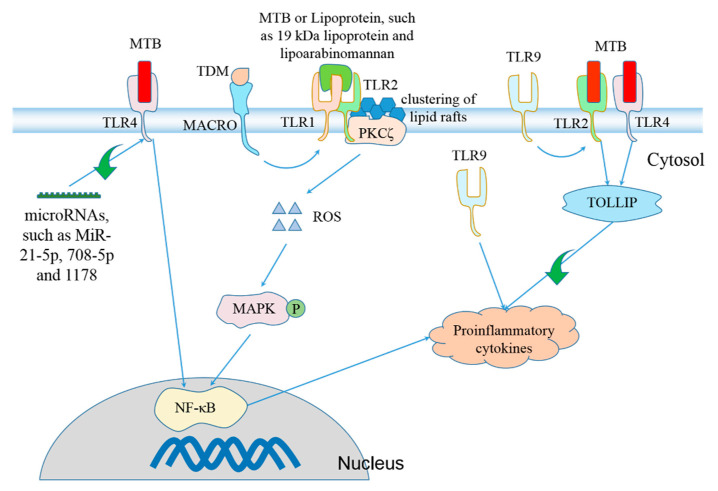
TLRs are involved in regulation of *Mycobacterium tuberculosis* (MTB)-induced production of proinflammatory cytokines. Stimulation with MTB [90], lipoarabinomannan [91] or 19 kDa lipoprotein from MTB [92] causes formation of TLR1/TLR2 heterodimer [93,94], inducing clustering of lipid rafts through interaction with protein kinase C ζ (PKCζ) and subsequent ROS formation [92]. Accumulation of ROS activates mitogen-activated protein kinases (MAPKs) [95,96], which promotes expression and secretion of proinflammatory cytokines via activation of NF-κB signaling pathway [97,98,99]. The class A scavenger receptor macrophage receptor with collagenous structure (MARCO) tethers trehalose 6,6’-dimycolate (TDM/cord factor) that is the predominant cell wall glycolipid of MTB to the macrophages to activate TLR2 [100]. Toll-interacting protein (TOLLIP) negatively regulates TLR2- and TLR4-mediated production of proinflammatory cytokines [101]. MTB stimulation activates TLR4 [81,102], which facilitates NF-κB-dependent production of proinflammatory cytokines [103]. TLR4 expression is negatively controlled by some microRNAs, including MiR-21-5p [104], MiR-708-5p [105] and MiR-1178 [106]. TLR9 cooperates with TLR2 to promote generation of proinflammatory cytokines [82].

Toll-like receptors (TLRs) are highly conserved pattern recognition receptors that sense specific invariant elements from pathogens and activate NF-κB signaling [76]. They are necessary for acute activation [76,77] and priming step of the NLRP3 inflammasome [78]. TLRs participate in macrophage activation upon MTB infection [79]. Multiple ligands expressed by MTB bind to TLRs, activating proinflammatory immune responses (Figure 1). TLRs play a vital role in host defense against MTB infection. TLR2 recognizes diacylated or triacylated lipoproteins by forming heterodimers with TLR6 or TLR1 [80]. TLR2^-/-^ mice are more susceptible following aerosol infection with 2000 CFU MTB per lung, and the serum level of proinflammatory cytokine IL-12p40 is lower than that of WT mice 10 days postinfection [81]. The role of TLR2 in protection against MTB infection was confirmed by Andre and colleagues’ study [82]. The innate immune system senses lipopolysaccharide (LPS) via TLR4 [83]. TLR4 knockout leads to decreased level of IL-12p40 in lung homogenate supernatant 4 weeks postinfection, higher mortality and shorter survival time after intranasal inoculation with 10^5^ or 5 × 10^5^ CFU [84] or aerosol infection with 2000 CFU MTB [85], but the Reiling and colleagues study showed that TLR4^-/-^ mice succumb to aerosol infection with 2000 CFU MTB with similar kinetics as WT mice [81]. TLR9 recognizes unmethylated CpG dinucleotides in microbial DNA sequences [86]. The genome of MTB possesses highly immunostimulatory CpG motifs [87]. DNA from MTB induces production of IL-12p40 and IL-6 in BMDCs and BMDMs. TLR9^-/-^ mice are more susceptible after aerosol infection with 50–100 or 500 CFU MTB, although only infection with the high dose causes higher bacterial burden in lungs. Compared to TLR2^-/-^ or TLR9^-/-^ mice, TLR2/9^-/-^ mice are more susceptible [82]. Deficiency of TLR1 surface expression coupled with specific genotypes is associated with susceptibility to TB [88]. TLR6^-/-^ mice display similar bacterial burden in lungs and spleens, mRNA expression of proinflammatory cytokines and pulmonary histopathology compared with WT mice [89].

**Figure 2:**
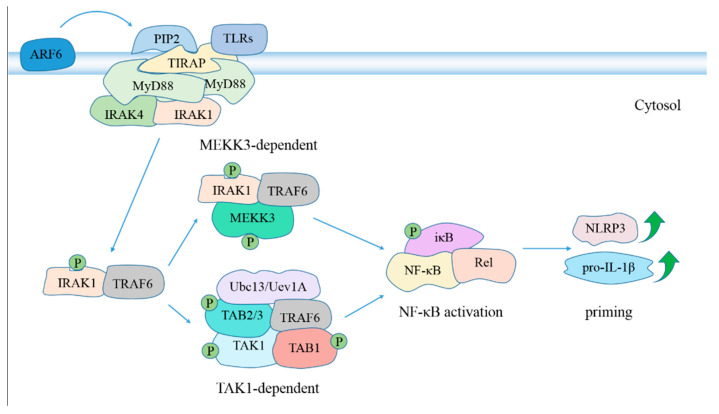
The role of MyD88 in NLRP3 inflammasome activation. Activated TLRs, including TLR2 [111,112], TLR4 [113,114], TLR5 [115], TLR2/6 heterodimer [116], TLR1/2 heterodimer [117] and TLR4/5 [118,119], facilitate activation of NF-κB signaling in a MyD88-dependent manner, which promotes NLRP3 inflammasome activation in the priming step [120]. ADP ribosylation factor 6 (ARF6) regulates synthesis of phosphatidylinositol 4,5-bisphosphate (PIP2) [121], which binds preferentially to Toll-interleukin 1 receptor (TIR) domain containing adaptor protein (TIRAP), provoking TIRAP transport to plasma membrane and subsequent MyD88 recruitment to activated TLR4 [122], TLR2 [123] and TLR5 [124]. TIRAP is dispensable for NF-κB activation following stimulation with high concentrations of TLR2 ligands [125]. MyD88 dimers recruit IL-1R–associated kinase 4 (IRAK4) and IRAK1 through its intermediate domain and N-terminal death domain, respectively [126,127]. This bridges the IRAK1 and IRAK4 kinase domains in close association, causing phosphorylation of IRAK1 by IRAK4 and subsequent IRAK1 autophosphorylation [128,129]. IRAK1 dissociates from MyD88 and TOLLIP after phosphorylation [130], and interacts with tumor necrosis factor receptor associated factor 6 (TRAF6) [128], leading to activation of NF-κB in TAK1-dependent and MEKK3-dependent pathways [131]. Abbreviation: MEKK3, MAPK/ERK kinase kinase 3; TAK1, transforming growth factor (TGF)-β-activated kinase 1; TAB1, TGF-β activated kinase 1 binding protein 1; Ubc13, ubiquitin-conjugating enzyme 13; Uev1A, Ubiquitin-conjugating enzyme variant 1A.

Myeloid differentiation factor 88 (MyD88), a general adaptor protein for the Toll/IL-1R family of receptors [107,108], is crucial for triggering macrophage effector mechanisms central to host defense against MTB infection via mediating inflammatory immune response. It contributes to upregulation of NLRP3 and IL-1β through activating NF-κB signaling, facilitating NLRP3 inflammasome activation (Figure 2). TNF-α release is abolished, and IL-12/IL-23p40 secretion is impaired in BMDMs from MyD88^-/-^ mice after MTB stimulation. Compared to that in the lungs of WT mice, the bacterial load in the lungs of gene-disrupted mice have 15-fold and 4600-fold increases at day 21 and 35 following aerosol infection with 100 CFU MTB, respectively. The lungs harbor massive cellular infiltrations and extensive necrosis of granulomas. MTB dissemination results in higher bacterial burden in the livers and spleens. MyD88^-/-^ mice succumb much earlier due to uncontrolled mycobacterial growth [109]. The roles of MyD88 in facilitating release of proinflammatory cytokines, restricting bacterial replication and prolonging survival were also confirmed by another study [110]. Absence of MyD88 provokes higher bacterial load, leading to increased numbers of neutrophils and macrophages, but does not influence antigen-specific activation of T cells and Th1 immune response induction. *M. bovis* BCG immunization 35 days prior to MTB challenge confers a substantial protection in MyD88^-/-^ mice from acute infection [110]. MyD88, rather than TLR2, TLR4 or TLR9, is critical for anti-mycobacterial effector responses. MyD88 knockout elicits higher bacteria load in lungs, spleens and livers, and lower levels of TNF-α and IL-12/IL-23p40 than TLR2, TLR4, TLR9 or TLR2/4/9 knockout [109].

Adaptor TIRAP mediates MyD88 transport to plasma membrane to mount an inflammatory response. However, association between Tirap polymorphism and TB risk are controversial, perhaps due to the different ethnicities. The heterozygous genotypes of Tirap C539T (also known as rs8177374 or S180L) in south Indian [132], G286A in China [133] and C558T in Vietnam [134] are associated with high risk for pulmonary tuberculosis (PTB), while the C539T variant in UK, Vietnam, several African countries [135], Colombia [136], Italy, Romania and Ukraine [137] was found to be a protective factor against PTB. Tirap polymorphisms in South Africa [138], Russia, Ghana, Indonesia [139], Colombia [140] and Zhengzhou, China [141] are not involved in TB susceptibility. In addition, Tirap^-/-^ mice possess the similar capacity to control acute MTB infection [142]. Liu and colleagues conducted a meta-analysis to evaluate the association between TIRAP C539T polymorphism and TB risk based on the data from 16 studies published from 2006 to 2013. The results indicates that TIRAP C539T is associated with decreased risk for PTB, especially in Europe [143].

MTB activates the NLRP3 inflammasome via several constituents, including ESX-1 secretion system [34], Rv1579c (also called EST12) [144], Rv0878c (also called PPE13) [145], the cell wall component mannosylated lipoarabinomannan [60] and dsRNA [64]. MTB damages phagosomal and plasma membranes during phagocytosis of bacteria, leading to K^+^ efflux and activation of NLRP3-dependent IL-1β release and pyroptosis [34]. ESAT-6 is a marker for mycobacterial viability and an ESX-1 substrate. It disrupts the host cell membranes by causing formation of pores ~4.5 nm in diameter [146]. MTB lacking ESAT-6 is unable to induce NLRP3 inflammasome activation, and treatment with purified MTB ESAT-6 triggers caspase-1 activation and IL-1β release. Additionally, ESAT-6 facilitates the delivery of immunostimulatory bacterial products such as AG85 into the cytosol, further augmenting NLRP3 inflammasome activation [60,64]. MTB induces IL-18 expression at both mRNA and protein levels via ESAT-6 in alveolar epithelial cells. Stimulation with ESAT-6 triggers ERK and p38 MAPK phosphorylation and production of ROS, which promotes IL-8 transcription and mRNA stability [147]. Stimulation with *M. bovis* BCG complemented with region of difference 1 (RD1), which encodes a part of the ESX-1 secretion system, induces IL-1β release [63]. Rv1579c, secreted from MTB H37Rv RD3, interacts with the receptor for activated C kinase 1 (RACK1) via its amino acid Y80 at the C-terminus, then recruits ubiquitin C-terminal hydrolase L5 (UCHL5) to deubiquitinate NLRP3, and finally activate the NLRP3 inflammasome [144].

In spite of the role of the NLRP3 inflammasome in host defense against MTB which is demonstrated by plenty of in vitro studies, in vivo studies show that only ASC mediates host protection during chronic MTB infection, while NLRP3 and caspase-1 are dispensable [35]. MTB bacterial burden in lungs and spleens, IL-1β and IL-1α concentrations in lung homogenates, the size, morphology and cellular composition of the lung lesions are not affected by NLRP3 absence following infection with virulent MTB via aerosol [63]. Nlrp3^-/-^ mice also have a similar survival profile to WT controls. Compared to WT mice, Caspase-1^-/-^ mice display similar levels of bacteria in the lungs and survival profile as well as even higher levels of IL-1β in lung homogenate extracts. ASC disruption leads to decreased survival time and fewer granulomas, although it has no effect on mycobacterial burden in the lungs [35]. Thus, Nlpr3^-/-^ and caspase-1^-/-^ mice have compensatory mechanisms of processing IL-1β and forming organized granulomas, and ASC is involved in host defense against MTB in NLRP3- and caspase-1-independent manners.

## 5. MTB and the AIM2 Inflammasome

If MTB infection activates or inhibits the AIM2 inflammasome is of debate. On the one hand, MTB residing in the phagosomes permeabilizes the phagosomal membrane early after infection via the ESX-1 secretion system, which results in release of phagosomal contents, including MTB and its DNA, into the cytosol [148]. How DNA is liberated from MTB is still unclear. Saiga and colleagues found that released DNA is sensed by and co-localized with AIM2, provoking AIM2 inflammasome activation. Compared to peritoneal macrophages from WT mice, cleavage of caspase-1 and expression of IL-1β and IL-18 at both the mRNA and protein levels are reduced in the cells from Aim2^-/-^ mice following infection with MTB [149]. *M. bovis*, a member of the MTB complex, is also able to cause TB in human beings. Its genome sequence is more than 99.95% identical to that of MTB [150]. Yang and colleagues found that *M. bovis* challenge induces upregulation of AIM2 at or after 24 hpi in J774A.1 macrophages and BMDMs. The siRNA-mediated knockdown of AIM2 expression impairs caspase-1 activation and IL-1β secretion, as well as release of lactate dehydrogenase (LDH) at 24 hpi in J774A.1 cells [151]. On the other hand, Shah and colleagues found that IL-1β release is inversely correlated with the virulence in mycobacterial species based on the detection of IL-1β levels in the culture supernatant following infection with *Mycobacterium smegmatis*, *Mycobacterium fortuitum*, *M. kansasii*, MTB H37Ra and MTB H37Rv. Aim2 deletion makes no change to IL-1β secretion in LPS-primed BMDCs at 16 hpi after challenging with MTB H37Rv. LPS-primed cells pretreated with MTB H37Rv, but not ESAT-6 deletion mutant, secrets less IL-1β and IL-18 in response to *M. smegmatis* or poly(dA:dT), indicating that virulent MTB strains inhibits AIM2-dependent IL-1β release [152]. These two different conclusions may result from the two following reasons: firstly, Shah and colleagues used LPS-primed BMDCs, while Saiga et al. and Yang et al. utilized the cells that have not been pretreated with LPS. MTB infection activates the NLRP3 inflammasome, and LPS is supposed to promote NLRP3-dependent IL-1β secretion for its function in priming, which is required for NLRP3 activation [51]. This may decrease the contribution of AIM2 to MTB-mediated IL-1β release. In addition, the priming step is dispensable for AIM2 inflammasome activation, and poly(dA:dT) is able to activate caspase-1 in an AIM2-dependent manner in the absence of LPS [51]. Whether MTB pretreatment induces reduced IL-1β release in response to only poly(dA:dT) is still unclear. *M. smegmatis* without LPS induces little IL-1β release in J774A.1 cells and BMDMs [153]. Thus, more evidence is needed to support that the MTB infection inhibits AIM2 inflammasome activation and resultant IL-1β release. Secondly, Saiga and colleagues used BMDCs, while BMDM and J774A.1 macrophages were used in the former two studies. In addition to AIM2, MTB DNA released into the cytosol can also be sensed by cyclic GMP-AMP synthase (cGAS) [154,155] and interferon-γ inducible protein 204 (IFI204) [154,156]. This triggers activation of type I IFNs signaling and autophagy.

AIM2 is indispensable for host defense against MTB infection. Aim2^-/-^ mice succumb within 7 weeks following intratracheal infection with MTB H37Rv, while WT mice are able to survive at least 8 weeks. At 4 weeks postinfection, higher bacterial load in the lungs and livers, more evident granulomatous changes and increased inflammatory cell infiltration in the lungs were found in Aim2^-/-^ mice. At 3 weeks after infection, the levels of IL-1β in BALF and IL-18 in serum from Aim2^-/-^ mice are lower than that from WT mice [149].

## 6. Regulation of Inflammasome Activation during MTB Infection

IFNs inhibit MTB-mediated inflammasome activation. Type I IFNs inhibits production of IL-1α and IL-1β in macrophages and DCs in lungs of MTB-infected mice [8]. cGAS generates the second messenger cGAMP after recognizing and binding MTB or *M. bovis* DNA released into the cytosol. Stimulator of interferon genes (STING) interacts with cGAMP, contributing to production of type I IFNs through activating interferon regulatory factor 3 (IRF3) [154,157]. Type I IFNs are detrimental for the control of MTB [158,159]. They play an inhibitory role in IL-1β production at its mRNA level. Addition of exogenous IFN-β or supplementation of culture medium with neutralizing antibody for IFN-α/βreceptor 2 (IFNABR2) affects the expression of IL-1β mRNA, rather than caspase-1 cleavage. *M. bovis* BCG does not trigger significant mRNA expression of type I IFNs [160]. Guarda and colleagues proposed that Type I IFNs inhibit inflammasome activation and IL-1β production through two independent mechanisms. On the one hand, Type I IFNs bind IFNAR, inducing secretion of anti-inflammatory cytokine IL-10. IL-10 interacts with its receptor IL-10R, decreasing the expression of pro-IL-1 at the protein level via activation of signal transducers and activators of transcription 3 (STAT3). The inhibitory effect of IFN-α or IFN-β on expression of pro-IL-1α and pro-IL-1β becomes less prominent in BMDMs isolated from Stat3^-/-^ or Il-10^-/-^ mice. Compared to control Stat3^flox/−^ BMDMs, the NLRP3 agonist aluminum slats-mediated caspase-1 cleavage is not altered in the presence of type I IFNs in Stat3^-/-^ cells. On the other hand, STAT1 is phosphorylated at tyrosine 701, which mediates inhibition of NLRP3-dependent caspase-1 activation. IFN-α or IFN-β fails to induce inhibition of activated caspase-1 in Stat1^-/-^ BMDMs in response to aluminum salts. IFN-β inhibits activation of the NLRP1b and NLRP3 inflammasomes, but not the AIM2 and IPAF inflammasomes. IFN-β inhibits caspase-1 activation following stimulation with NLRP3 inducers, including monosodium urate crystals, asbestos, nigericin, ATP and *Candida albicans*, and the NLRP1b inducer *Bacillus anthracis* lethal toxin, rather than the AIM2 agonist poly(dA:dT) or the IPAF agonist *Salmonella typhimurium*, though amounts of mature form and precursor of IL-1β are diminished in all cases [161]. IL-1 and type I IFNs mutually regulate each other via prostaglandin E2 (PGE2) to control the balance. Ifnar1 knockout results in increased PGE2 and IL-1β in BALF, and addition of exogenous IFN-β to MTB-infected BMDMs or human MDMs reduces PGE2. Knockout of Il1r1 or IL-1α/β enhances IFN-α and IFN-β at both the mRNA and protein levels [162]. CD4^+^ T cell-derived IFN-γ plays a protective role in MTB control [163]. It inhibits expression of IL-1α and IL-1β only in inflammatory monocytes [8] and does not influence pro-IL-1 expression as well as caspase-1 activation and IL-1β maturation in BMDMs [161]. Meanwhile, IFN-γ facilitates iron export through control of the expression of iron regulatory proteins hepcidin and ferroportin, and prevents MTB-induced intracellular iron sequestration, retarding the bacterial growth by decreasing iron availability [164].

## 7. Concluding Remarks

Remarkable advances in MTB–host interaction have been made. Many studies identified the roles of certain cytokines in host defense against MTB infection. IL-1 plays a protective role, while type I IFNs have a detrimental effect. Most reports demonstrated that MTB triggers NLRP3 inflammasome activation and subsequent maturation and release of proinflammatory cytokines via ESX-1 secretion system and its substrate ESAT-6 in vitro, but NLRP3 and caspase-1 are dispensable for control of MTB in vivo. AIM2 facilitates to restrict MTB replication both in vitro and in vivo. Type I IFNs suppress IL-1β activity through interaction with IFNAR. However, the mechanisms by which IL-1β is regulated is still unclear. AIM2 is indispensable for activities of IL-1β and IL-18, but caspase-1 does not contribute to higher levels of IL-1β in vivo, implicating that AIM2 exerts its protective function in a caspase-1-independent manner after sensing MTB DNA released into the cytosol. Exploration of the role of inflammasome in host defense against MTB infection, especially the regulation of IL-1, contributes to a better understanding of MTB–host interaction and provides potential therapeutic targets for treating TB. 

## Data Availability

Data sharing not applicable.
